# Abemaciclib-Associated Panniculitis With Fibrosis: Expanding the Dermatologic Spectrum of Cyclin-Dependent Kinase 4/6 Inhibitors

**DOI:** 10.7759/cureus.83380

**Published:** 2025-05-02

**Authors:** Varsha Pujala, Shravya Balmuri, Tharwat Ghattas, Millicent Amankwah, Dheeraj kumar Posa

**Affiliations:** 1 Internal Medicine, Louisiana State University Health Sciences Center, Shreveport, USA; 2 Hematology and Medical Oncology, Louisiana State University Health Sciences Center, Shreveport, USA

**Keywords:** abemaciclib, cdk4/6 inhibitors, chronic panniculitis with fibrosis, dermatologic side effects, panniculitis

## Abstract

Abemaciclib, a cyclin-dependent kinase 4/6 (CDK4/6) inhibitor, has significantly improved outcomes for patients with hormone receptor-positive/human epidermal growth factor receptor 2-negative (HR+/HER2-) metastatic breast cancer, yet its dermatologic side effects remain incompletely characterized. We report a case of a 55-year-old woman on abemaciclib and fulvestrant who developed painful, erythematous nodules that progressed to hyperpigmented plaques with skin retraction, including on the breasts. A biopsy revealed chronic panniculitis with fibrosis, a histopathologic finding not previously associated with CDK4/6 inhibitors. Despite these cutaneous changes, abemaciclib was continued without dose modification, and the lesions remained stable without ulceration. This case expands the known spectrum of skin toxicities linked to CDK4/6 inhibition. It underscores the importance of recognizing panniculitis as a potential immune-mediated adverse event, which, in select cases, may be managed conservatively without interrupting oncologic therapy.

## Introduction

Abemaciclib, a cyclin-dependent kinase (CDK) 4/6 inhibitor, has emerged as a cornerstone therapy for hormone receptor-positive/human epidermal growth factor receptor 2-negative (HR+/HER2-) metastatic breast cancer; it acts by targeting CDK4 and CDK6 proteins, halting cell cycle progression at the G1 phase, thereby inhibiting cancer proliferation and disease progression. Abemaciclib is the first CDK inhibitor to be approved for use as a standalone treatment or to be used in combination with fulvestrant for patients who have progressed on hormone therapy [1].

 Abemaciclib has changed the therapeutic landscape of HR-positive breast cancer; it is accompanied by an array of adverse effects, among which diarrhea is prominent, necessitating dose adjustment or therapy termination; other common toxicities include neutropenia, nausea, and hepatotoxicity. Dermatologic toxicities, though present, are underreported and insufficiently understood [2]. This case report delves into rare and clinically intriguing manifestations of abemaciclib-induced chronic panniculitis with fibrosis.

Panniculitis is a condition characterized by inflammation of the subcutaneous fat layer, usually appearing as erythematous, tender bumps or plaques that can be confirmed by histopathological studies.

## Case presentation

A 55-year-old African-American woman with a past medical history of anemia, chronic obstructive pulmonary disease (COPD), diabetes mellitus, hypertension, and mixed hyperlipidemia was diagnosed with ER+/PR+/HER2- breast cancer in 2012. She underwent lumpectomy, chemotherapy (AC-T), and 10 years of endocrine therapy (tamoxifen, letrozole). In January 2023, a sacral mass was incidentally found after a motor vehicle accident, and a biopsy confirmed metastatic breast cancer. She was initiated on fulvestrant and abemaciclib in March 2023. By May 2024, she developed multiple painful, erythematous subcutaneous nodules on her extremities and trunk. She had pruritus initially when the lesions appeared, but then stopped after a while. Her pain was minimal and controlled; she did not get anything for pain. Over time, lesions were seen on both breasts, evolving into hyperpigmented lesions with skin retraction.

**Figure 1:**
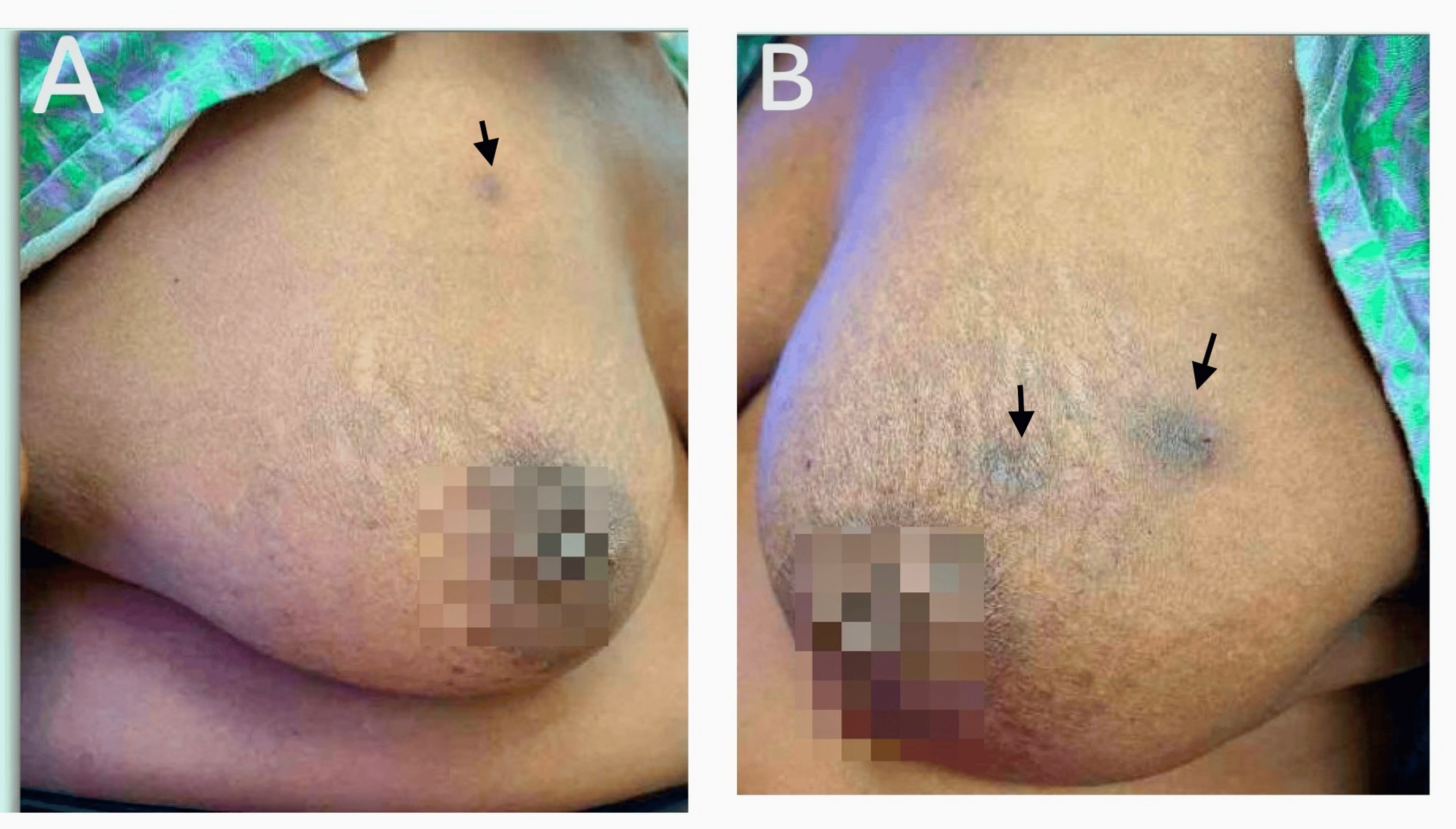
(A) Image of the right breast with hyperpigmented lesion. (B) Image of the left breast showing two hyperpigmented lesions

Since starting on fulvestrant and abemaciclib, her labs showed mild leukopenia (between 2.7 and 3.35 K/uL, normal range: 3.9-12.7K/uL). Her vitamin D, B12, and TSH levels were within normal limits. Figure 1 illustrates the clinical appearance of the breast lesions, showing extensive hyperpigmentation and skin tethering.

An excisional biopsy of the previous partial mastectomy site and punch biopsies of chest lesions were done in December 2024, revealing chronic panniculitis with fibrosis without evidence of malignancy.

**Figure 2:**
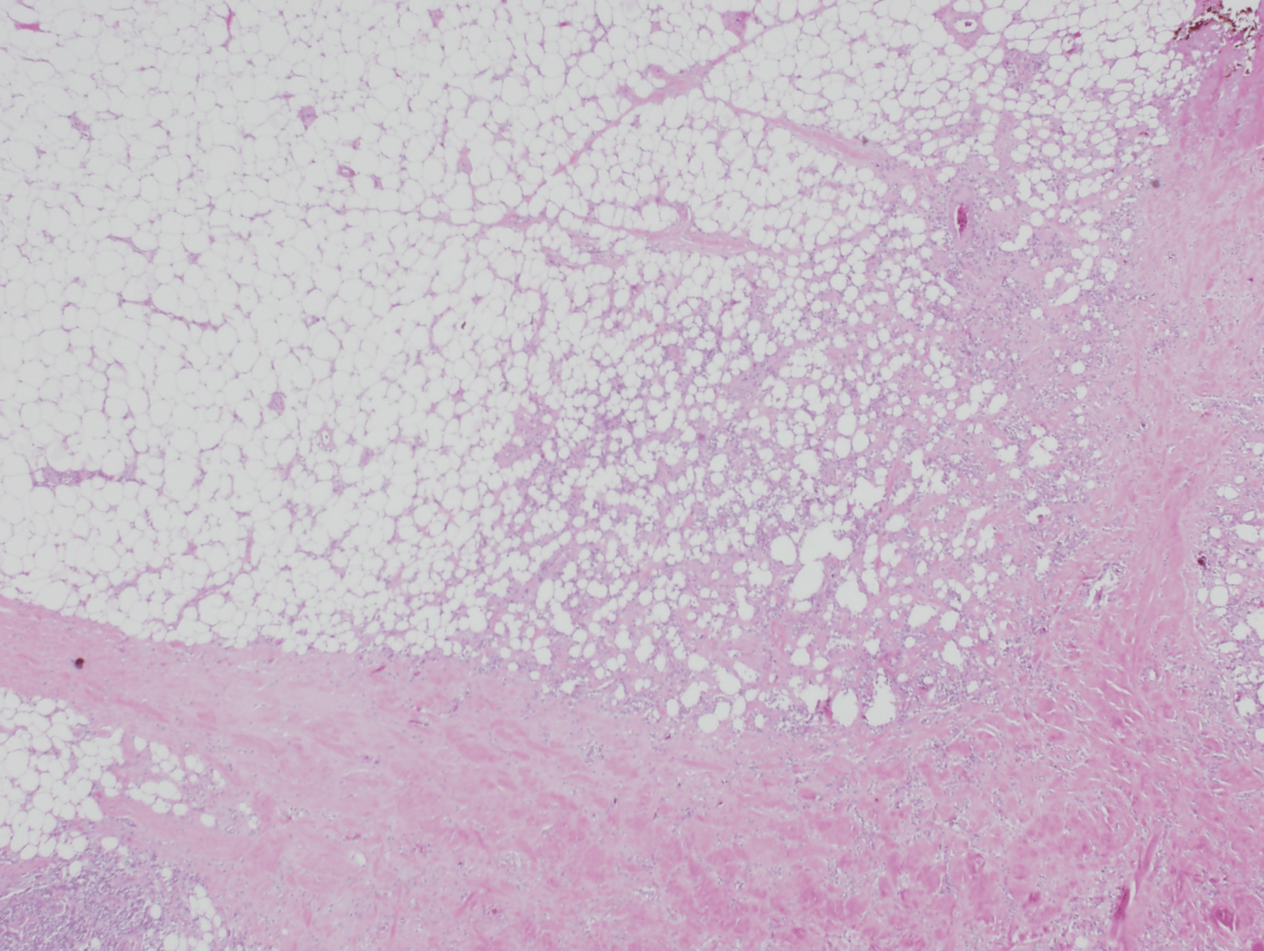
Photomicrograph of the skin lesion showing variable involvement ranging from normal fat, inflammation, and fibrosis

**Figure 3:**
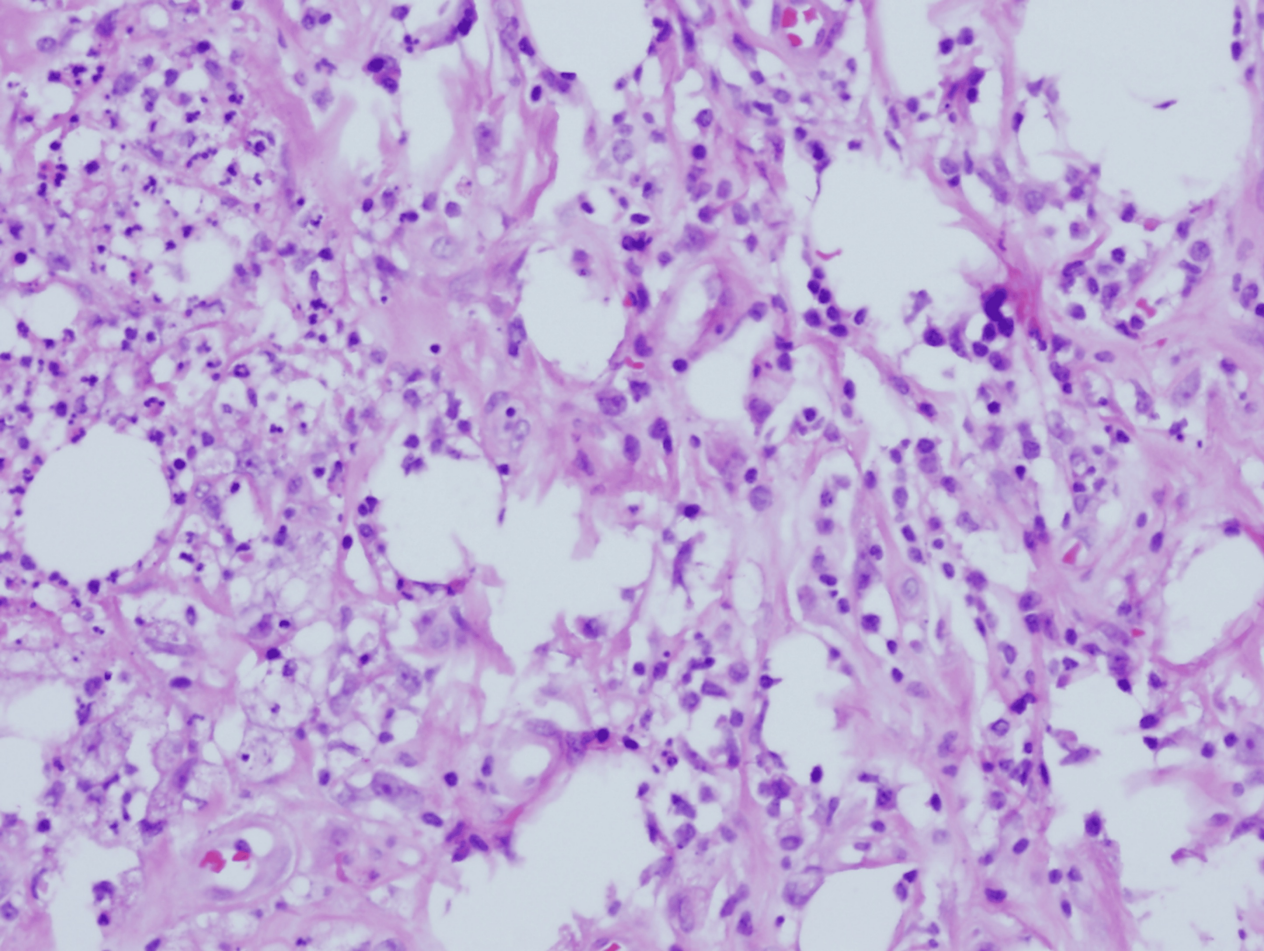
Histopathology of the skin lesion (H&E staining) showing chronic panniculitis with fibrosis

As seen in Figure 2, the photomicrograph highlights the heterogeneous involvement across the lesion, ranging from preserved adipose tissue to regions of active inflammation and dense fibrotic changes. Figure 3 provides a closer histologic view with H&E staining, demonstrating lobular panniculitis with chronic inflammatory infiltrate and fibrosis.

We opted to continue abemaciclib after discussing with the patient, without any dose modification, given its therapeutic benefit. The lesions remained stable without ulceration.

## Discussion

Panniculitis is a condition characterized by inflammation of the subcutaneous fat layer, usually appearing as erythematous, tender bumps or plaques, mainly on the lower legs but sometimes elsewhere on the body, mimicking subcutaneous lymphoma, metastatic cancer, or autoimmune processes [3]. Clinical evaluation, detailed history, and thorough histopathological examination are needed to confirm the diagnosis [4]. Panniculitis is broadly categorized into septal and lobular forms, which can be associated with or without vasculitis. Panniculitis following the use of biological therapeutic agents, as in our case, falls under septal panniculitis with the absence of vasculitis [4].

Among advances in oncology, CDK4/6 inhibitors - palbociclib, ribociclib, and abemaciclib - have proven instrumental in revolutionizing the standard of care for HR+/HER2- metastatic breast cancer, the common subtype. These drugs selectively inhibit the CDK4 and CDK6 complex, thereby halting the cell cycle progression from G1 to S phase, significantly improving progression-free survival when combined with endocrine therapy [5].

Dermatological toxicities are among the many side effects associated with CDK4/6 inhibitors, notwithstanding their therapeutic advantages. In as many as 15% of cases, cutaneous manifestations manifest as anything from severe immune-mediated reactions to nonspecific rashes [6]. The following are reported cutaneous adverse effects linked to CDK4/6 inhibitors, according to a review of the literature: alopecia, bullous skin rashes, Stevens-Johnson syndrome (SJS), toxic epidermal necrolysis (TEN), radiation recall dermatitis (RRD), radiation dermatitis, cutaneous leukocytoclastic vasculitis, Henoch-Schönlein purpura (HSP), subacute cutaneous lupus erythematosus (SCLE), chronic cutaneous lupus erythematosus, xerosis, vitiligo-like lesions, histiocytoid Sweet syndrome (HSS), and erythema dyschromicum perstans (ashy dermatosis) [6,7].

Panniculitis has not yet been identified as an adverse effect of CDK4/6 inhibitors. Our case makes it essential to recognize panniculitis as a possible side effect of abemaciclib. These dermatologic toxicities may be caused by direct impacts on skin cells or immunological dysregulation, while the precise mechanisms remain unknown. Immune modulation and inflammatory reactions brought on by CDK4/6 inhibition are examples of potential mechanisms that could lead to panniculitis.

CDK4/6 inhibitors, including abemaciclib, can induce a range of dermatologic adverse effects [8], and our case suggests that chronic panniculitis may be one such manifestation, potentially triggered by drug-induced immune modulation. Topical steroids and/or emollients are used to manage mild cutaneous adverse effects, and systemic steroids or dose reduction are used in severe reactions [8]. Our patient's symptoms resolved spontaneously over time without any specific intervention, allowing for the uninterrupted continuation of abemaciclib therapy. Early recognition and appropriate management of panniculitis must be emphasized in patients receiving CDK4/6 inhibitors to ensure optimal oncologic outcomes without unnecessary treatment discontinuation [8-10].

## Conclusions

This case highlights chronic panniculitis with fibrosis as a novel adverse effect of abemaciclib, expanding the known dermatologic profile of CDK4/6 inhibitors. Early recognition can prevent misdiagnosis as metastatic disease and unnecessary treatment discontinuation. Further studies are needed to characterize the incidence, mechanism, and optimal management of CDK4/6 inhibitor-associated panniculitis.

## References

[1] Sledge GW Jr, Toi M, Neven P (2017). MONARCH 2: abemaciclib in combination with fulvestrant in women with HR+/HER2- advanced breast cancer who had progressed while receiving endocrine therapy. J Clin Oncol.

[2] Rugo HS, Huober J, García-Sáenz JA (2021). Management of abemaciclib-associated adverse events in patients with hormone receptor-positive, human epidermal growth factor receptor 2-negative advanced breast cancer: safety analysis of MONARCH 2 and MONARCH 3. Oncologist.

[3] Lee J, Sathe NC (2024). Dermatopathology evaluation of panniculitis. StatPearls.

[4] Wick MR (2017). Panniculitis: a summary. Semin Diagn Pathol.

[5] Silvestri M, Cristaudo A, Morrone A (2021). Emerging skin toxicities in patients with breast cancer treated with new cyclin-dependent kinase 4/6 inhibitors: a systematic review. Drug Saf.

[6] Raschi E, Fusaroli M, La Placa M, Ardizzoni A, Zamagni C, Poluzzi E, De Ponti F (2022). Skin toxicities with cyclin-dependent kinase 4/6 inhibitors in breast cancer: signals from disproportionality analysis of the FDA Adverse Event Reporting System. Am J Clin Dermatol.

[7] Sibaud V, Sollena P (2023). Dermatologic toxicities to inhibitors of cyclin-dependent kinases CDK 4 and 6: an updated review for clinical practice. Ann Dermatol Venereol.

[8] Sollena P, Vasiliki N, Kotteas E (2023). Cyclin-dependent kinase 4/6 inhibitors and dermatologic adverse events: results from the EADV Task Force “dermatology for cancer patients” international study. Cancers (Basel).

[9] Alsatti H, AlMalki B, Alghamdi Y (2024). Abemaciclib-associated skin, hair, and nail toxicities: a case report. Cureus.

[10] Amigo M, Hoffman K, Chung C (2022). Presentation and management of diverse cutaneous reactions after cyclin-dependent kinase 4/6 inhibitor use. J Am Acad Dermatol.

